# Radiation Exposure to Staff in Intensive Care Unit with Portable CT Scanner

**DOI:** 10.1155/2016/5656480

**Published:** 2016-07-31

**Authors:** Zhichao Xie, Xuelian Liao, Yan Kang, Jiangqian Zhang, Lingli Jia

**Affiliations:** Department of Critical Care Medicine, West China Hospital, Sichuan University, Chengdu 610041, China

## Abstract

*Background.* Bedside radiological procedures pose a risk of radiation exposure to ICU staff. The perception of risk may increase the degree of caution among the health care staff and raise new barriers preventing patients from obtaining prompt care.* Objective.* The aim of this study was to estimate the annual cumulative radiation dose to individual ICU staff.* Methods.* In this prospective study, forty subjects were required to wear thermoluminescent dosimeter badges during their working hours. The badges were analyzed to determine the exposure after 3 months.* Results.* A total of 802 radiological procedures were completed at bedside during the study period. The estimated annual dosage to doctors and nurses on average was 0.99 mSv and 0.88 mSv (*p* < 0.001), respectively. Residents were subjected to the highest radiation exposure (1.04 mSv per year, *p* = 0.002). The radiation dose was correlated with day shift working hours (*r* = 0.426; *p* = 0.006) and length of service (*r* = −0.403; *p* < 0.01).* Conclusions.* With standard precautions, bedside radiological procedures—including portable CT scans—do not expose ICU staff to high dose of ionizing radiation. The level of radiation exposure is related to the daytime working hours and length of service.

## 1. Introduction

Radiological procedures have become an important part of the management of critically ill patients in the intensive care unit. The procedures are used for diagnostic and therapeutic purposes, such as assessing the condition of heart, lung, or brain, and confirming the position of devices such as endotracheal tubes and central venous catheters. They are often performed at the bedside because transportation can be hazardous for critically ill patients [1]. However, the ionizing radiation from bedside radiological procedures poses a risk of radiation exposure to ICU personnel. Although the reported scattered radiation was minimal in the ICU ward [2, 3], no radiation dose can be considered safe. Epidemiological data indicate that exposure to even low-dose radiation may result in solid cancers and leukemia [4–6]. Therefore, the risk of cumulative radiation dose is a cause for concern among the ICU staff, especially with the increasing popularity of bedside CT scans in ICU wards.

Studies focus on the radiation dose to medical employees performing orthopedic surgery or interventional cardiology [7, 8]. However, few studies [2, 3, 9] investigate the radiation exposure in ICU ward, and none of them included the exposure from usage of portable CT scanner. CT scans involve larger radiation doses compared to conventional X-ray imaging procedures. The radiation dose to an adult's brain in a typical head CT is about 4000 times the dose delivered in a dental radiography [10]. The risk of exposure to scattered X-rays from CT scans is higher than conventional X-ray imaging procedures with the rotation of the X-ray tube during scanning. Therefore, increased attention should be paid to the scattered X-rays from CT scans, especially in the ICU ward without adequate protection.

The radiation dose range in both conventional X-ray imaging procedures and portable CT scans to ICU staff has not been reported. Most health care professionals are aware of the fact that all sources of radiation are harmful and stay as far away as possible from the machines while scanning [11]. Excessive precaution may cause damage to the health of critically ill patients. The objective of this study was to estimate the annual cumulative radiation dose received by ICU staff where bedside CT scan is available.

## 2. Materials and Methods 

In this prospective, observational study conducted in a 50-bed medical ICU of an academic teaching hospital from July 1 to October 1, 2014, 40 participants (including 18 nurses, 14 residents, and 8 attending doctors) were randomly selected from the volunteers who were full-time staff in the ICU. The residents performed more clinical tasks than others, and the nurses worked in 8-hour shifts.

The bedside radiological procedures were performed by radiologists from the Department of Radiology. The ICU staff were not directly involved in the procedures and were required to stay at least 4 m away from the scanner during the procedure. In addition to the lead curtains around the CT scanner, lead shielding was used to block the scattered X-rays. No additional personal protective devices were used because of their heavy weight and inconvenience.

Thermoluminescent dosimeters (TLDs) were used to measure the cumulative radiation dose to the staff. The ionizing radiation was absorbed and stored in a crystal inside the TLD badge when the X-rays pass through the badge. When heated in the detector, the crystal emits visible light, which was measured to calculate the cumulative dose of ionizing radiation. The amount of light emitted depends on the level of radiation exposure. The dosimetric range of TLDs is 10 *μ*Gy to 10 Gy. During the study period, the participants wore specifically assigned TLD badge in the ward and were requested to remove the badge when leaving the ward. Three badges were maintained as background control in the office area where no radiological procedures were performed. Each of the badges was numbered for identification and analysis. The badges were then processed to determine the amount of radiation exposure by a qualified independent third party who was blinded to this study. The estimated annual dose was calculated by expanding these data four times.

The bedside radiographs were performed using a mobile X-ray system (MobileDaRt Evolution, Shimadzu Corporation, Japan). An 8-slice portable CT scanner (CereTom, NeuroLogica Corporation, USA) was used for neurological CT scans. The TLD signal was read using a Harshaw 6600 Plus Automated Reader (Thermo, USA).

Data are presented as mean ± SD for normally distributed variables and medians and interquartile ranges for skewed variables. Radiation doses to participants were analyzed using one-way ANOVA. Correlations between two quantitative variables were determined by Pearson correlation analysis. Statistical analysis was performed using SPSS software, version 19.0 (IBM, USA).

## 3. Results

A total of 802 radiological bedside procedures were completed during the study period, which included 613 chest X-rays and 189 head CT scans. The radiation dose of a regular chest radiograph was 0.130 mGy, and the dose-length product of regular CT scan was 660.128 mGy·cm per exam (16 rotations × 2 seconds per rotation × 7 mA = 224 mAs).

The mean age of all participants was 29.7 years (range: 23 to 46 years). The mean BMI was 20.5 kg/m^2^ (range: 16.5 to 24.8 kg/m^2^). Forty-five percent of the participants were males. Most of the female employees (77%) were aged less than 35 years. Nurses spent 40 ± 0.45 hours per week working in the ward, while the workweek of doctors was 54.5 ± 0.89 hours (Table 1).

**Figure 1:**
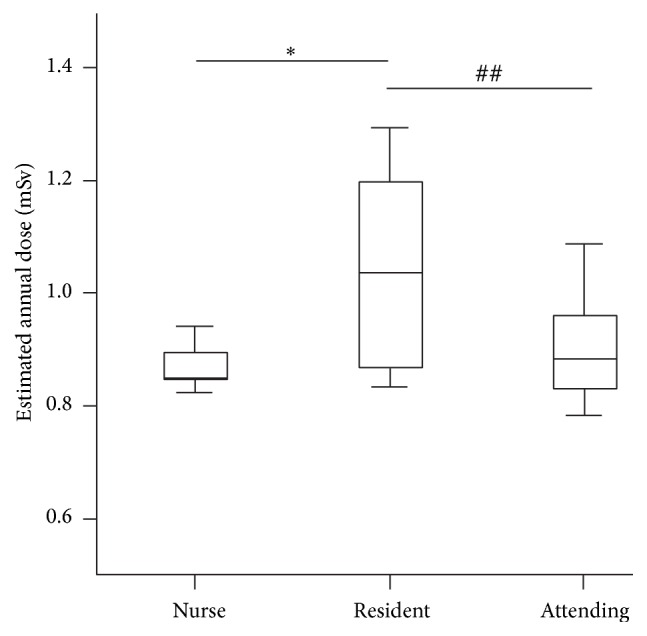
The estimated annual doses to all participants. *∗*, post hoc test between resident and nurse, *p* < 0.001; ##, post hoc test between attending and resident, *p* = 0.012.

**Figure 2:**
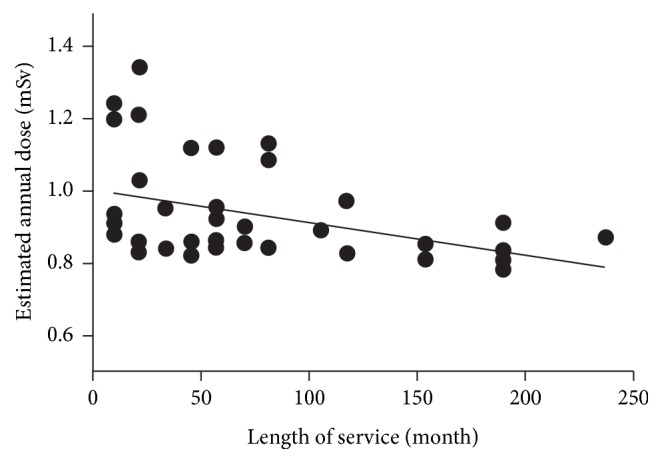
Relationship of radiation dose to length of service. A moderate negative correlation has been shown between radiation dose and length of service (the Pearson correlation coefficient was −0.403, *p* < 0.01).

The baseline radiation recorded by control badges during the three months was 0.21 ± 0.01 mGy, and all other values were reported above this baseline (background radiation has been deducted). A significantly higher dose was recorded in the doctors than in the nurses (0.24 ± 0.04 mSv versus 0.22 ± 0.01 mSv, *p* < 0.001). The mean estimated annual doses to doctors and nurses were 0.99 mSv and 0.88 mSv (*p* < 0.001), respectively. Residents were subjected to the highest radiation exposure, with a radiation dose of 1.04 mSv per year (*p* = 0.002) (Table 2).  Figure 1 illustrates the estimated annual dose to all participants. A moderate correlation was seen between radiation dose and day shift working hours (*r* = 0.426, *p* = 0.006). Radiation dose was also negatively associated with length of service since appointment in this hospital (*r* = −0.403, *p* < 0.01) (Figure 2).

## 4. Discussion

Ionizing radiation, such as X-rays, creates ions by knocking electrons out of their orbits. The interaction between X-rays and water molecules results in hydroxyl radicals that induce strand breaks and base damage to nearby DNA. X-rays also ionize DNA directly. The occasional errors during DNA repair process result in point mutations, chromosomal translocations, and gene fusions [10]. These changes are closely linked to the occurrence of cancer. The likelihood of cancer in people who are exposed to radiation is proportional to the radiation dose. Additionally, radiation may cause nonneoplastic effects such as genetic mutations and developmental malformations in children whose mothers were exposed to radiation during pregnancy [12]. Two large-scale studies [4, 6] of numerous radiation workers in the nuclear industry have shown that an excess risk of cancer exists, even with low-dose chronic exposure to ionizing radiation. Therefore, no radiation dose can be considered safe. It is widely accepted that unnecessary exposure should be avoided to keep the received radiation dose as low as reasonably achievable (ALARA principle).

The International Commission on Radiological Protection (ICRP) [13] published recommendations for the appropriate dose limits of radiological protection. The maximum annual permissible occupational upper dose limits are 20 mSv for body, 150 mSv for the thyroid or eyes, and 500 mSv for the hands or skin. Routine personal dose monitoring is recommended if the cumulative dose is over 30% of these limits. Dose limits for the public are only 5–10% of the occupational limits (i.e., 1 mSv for the body, 15 mSv for the thyroid or eyes, and 50 mSv for the skin). It is more appropriate to classify ICU staff as occupational based on the recommendations of ICRP. However, according to the recommendations [13], fetuses should not be exposed to radiation doses more than 1 mSv/a irrespective of the mothers' profession. Therefore, it is necessary that pregnant ICU employees should be classified and protected as the public.

Our data show that the estimated annual dose to staff is below the limit (0.94 mSv versus 20 mSv). Although 8 doctors (36.4%) and 1 nurse (5.6%) received effective dosage exceeding 0.25 mSv during three months, it seems unlikely that ICU staff would be exposed to an annual dose over the ICRP recommended limit or 30% of the limit in the next few years. At the current level of protection, the cumulative radiation exposure to ICU staff was minimal, within the limit, and acceptable. The radiation of portable radiological procedures should not be overemphasized even in the ICUs where bedside CT scans are frequently performed.

Although there is no evidence supporting routine personal monitoring of all ICU staff, monitoring a small number of them is highly recommended, because many ICU employees are young women who may be pregnant with or without noticing and there is little disadvantage in monitoring at least a few staff at risk of exposure, particularly with the increased usage of bedside radiation in ICU ward.

Poor understanding of radiation and its hazards contributes to the fear of radiological procedures among the ICU staff. The relationship between radiation intensity and distance follows the inverse square law, meaning that if the staff member doubles his/her distance, he/she should receive a quarter dose. Therefore, they prefer to keep a far distance and discontinue to monitor patients during the procedures to reduce radiation exposure. Usually, patients undergoing radiological procedures and other patients nearby are left alone for two to three minutes or longer, which may expose patients to danger under specific circumstances. Nurses who left may fail to diagnose patients' accidental disconnection from mechanical ventilation devices, which can increase length of hospital stay or even be fatal. Recently, Dianati and colleagues [11] investigated intensive care unit nurses and found that they have limited knowledge of radiation safety, exposure, and protection. The knowledge of the ICU staff needs to be updated to enable appropriate protection and safety measures to dispel anxiety. With the increased number of radiation procedures performed, the increased understanding of radiation not only contributes to clinical success but also ensures patient safety.

As radiologic procedures are usually performed during the daytime, it is reasonable to correlate day shift with radiation exposure. The average dose of exposure was greater among the doctors compared with the nurses. Physicians, especially the residents, spent more time in the ward and were more likely to present during a bedside radiologic procedure because most of their working time involved day shift.

A moderate negative correlation was shown between radiation dose and length of service. Recently, a national survey [14] in South Korea investigated the occupational radiation exposure among radiologists. Similar relationship was found between higher radiation dose and new employees in their survey. New employees usually take on more work to do and spend more time in the workplace. They could also have limited knowledge regarding hazards of radiation, amount of environmental radiation of each radiological examination, and radiation protection strategies. More training should be conducted for new employees to enhance their knowledge of radiation safety and to regulate their behaviour towards portable radiologic procedures.

The level of exposure to scattered X-rays was less than 0.6 mSv per year in a 10-bed trauma intensive care unit (TICU) involving about 500 radiologic procedures in a month [3]. CT scans and fewer radiographs (4 versus 50 procedures per bed per month) were performed in our ICU. The radiation level in our study was higher than in the TICU due to exposure predominantly to the scattered X-rays from bedside CT.

The study limitations were related to performance of the procedure only in one medical ICU and the small sample size. Furthermore, the number and types of bedside radiological procedures varied each month according to the patients admitted to ICU, leading to differences in the overall radiation exposure each month. However, we estimated the annual radiation dose using the three-month data. Future studies with larger sample size and longer duration may overcome these limitations.

## 5. Conclusions

The study findings indicate that, with standard safety precautions, bedside radiological procedures—including portable CT—do not expose ICU staff to high doses of ionizing radiation. The level of radiation exposure is related to working hours during the day and the length of service. Currently, according to the level of effective dosage received and the increasing usage of bedside radiological procedures in ICU, monitoring a small number of professionals is highly recommended for measures to protect pregnant staff in case of radiation overdose.

## Figures and Tables

**Table 1:** Characteristics of all participants.

Occupation	Total (40)	Nurse (18)	Resident (14)	Attending (8)
Age (y)	29.7 ± 6.1	27.1 ± 3.9	27.4 ± 3.3	39.4 ± 4.0
Male	18 (45%)	4 (22%)	9 (64%)	5 (63%)
Female younger than 35 years of age	17 (77%)	12 (86%)	5 (100%)	0
BMI (kg/m^2^)	20.5 ± 2.0	20.2 ± 2.2	20.4 ± 1.5	21.5 ± 2.3
Workweek (hour)	—	40 ± 0.45	55 ± 0.59	53.5 ± 0.33
Day shift (hour)	—	22.5 ± 0.45	41 ± 0.59	39.5 ± 0.33
Length of service (month)	57 (21,99)	57 (21,69)	39 (18,60)	153 (108,189)

**Table 2:** Radiation dose to all of the roles.

Occupation	Radiation dose during 3 months (mSv)	Estimated annual dose (mSv)	*p* value^*∗*^
Nurse (18)	0.22 ± 0.01	0.88 ± 0.05	*p* < 0.001^#^
Doctor (22)	0.24 ± 0.04	0.99 ± 0.16	
Resident (14)	0.26 ± 0.04	**1.04 ± 0.18** ^*∗∗*^	*p* < 0.001^##^
Attending (8)	0.22 ± 0.02	0.90 ± 0.10	*p* = 0.012^†^
Total (40)	0.23 ± 0.03	0.94 ± 0.14	*p* = 0.002^††^

*∗*: comparison of estimated annual doses.

*∗∗*: the estimated annual dose to resident is higher than the limit ICRP recommended for the public.

#: *t*-test between nurse and doctor.

##: post hoc test between resident and nurse.

†: post hoc test between attending and resident.

††: ANOVA test among nurse, resident, and attending.
